# Commentary: Potentially inappropriate medication among older patients with diabetic kidney disease

**DOI:** 10.3389/fphar.2023.1210331

**Published:** 2023-06-29

**Authors:** M. I. Danjuma, R. Sayed, I. Elzouki

**Affiliations:** ^1^ Department of Internal Medicine, Hamad Medical Corporation, Doha, Qatar; ^2^ Department of Internal Medicine, Weill Cornell College of Medicine, New York, NY, United States; ^3^ Department of Internal Medicine, Weill Cornell College of Medicine, Doha, Qatar; ^4^ Department of Internal Medicine, Qatar University College of Medicine, Doha, Qatar; ^5^ NHS Grampian (Dr. Grays Hospital), Elgin, United Kingdom

**Keywords:** drug–drug interaction, polypharmacy, potentially inappropriate medication (PIM), adverse drug reaction, chronic kidney disease

## 1 Introduction

Wang et al. (2020)’s recent report on the burden and morbidity consequences of potentially inappropriate medications (PIMs) in a select cohort of type 2 diabetic mellitus (T2DM) patients was both instructive and an “inflection point” in the characterization of the clinical phenotype of this rising morbidity. They found an overall prevalence of 67.5% of PIMs amongst a randomly selected cohort of *n* = 186 T2DM patients, with the odds of having a PIM phenotype rising 4-fold amongst those hospitalized with concomitant polypharmacy (American Geriatrics Society, 2019; Wang et al., 2020). This report significantly advances the narrative regarding the evolving relationship between PIMs and polypharmacy. Downstream adverse consequences of polypharmacy are a “legion,” but the most serious of these include PIMs, and bidirectional interactions amongst others (Alves-Conceição et al., 2019). Whilst these relationships have since been extensively studied and reported in the general population (Richardson et al., 2015), there remains a paucity of studies purposefully set out to examine key themes of this relationship in patients with chronic kidney disease (CKD). These include the prevalence of polypharmacy as well as the global array of factors impacting this (American Geriatrics Society, 2019). Indeed, it is out of the recognition of this significant gap in our understanding that we recently reported the first systematic examination of the clinical burden as well as socio-demographic factors that globally drive this in patients with chronic kidney disease (Naseralallah et al., 2023). We found the overall pooled prevalence of polypharmacy amongst patients with CKD was 69% (95% CI: 49%–86%) (I^2^ = 100%, *p* < 0.0001), with a proportionately higher prevalence in North America and Europe as compared to Asia (American Geriatrics Society, 2019; Naseralallah et al., 2023; Chowdhury et al., 2017).

Wang et al. (2020)’s PIM estimate of 67.5% is consistent with that reported from recent studies, although those exploring larger patient populations such as Roux-Marson et al. (2020), found relatively lower PIM prevalence estimates. These include Jones et al. (2020)’s estimate of 13% and Roux-Marson et al. (2020)’s case burden of 45.1% among patients with CKD. The exact nexus between polypharmacy and PIMs are still evolving, and estimates reported today are likely to change as more and more “candidate” drugs receive requisite market authorizations (MA). This is so because what constitutes “medication propriety” is both disease and patient demography specific (Gallagher et al., 2011). What is potentially inappropriate for patients with specific organ morbidities (such as CKD, heart failure, and stroke), for example, may for all intents and purposes be perfectly “reasonable” for patient cohorts with alternative diagnoses. Even amongst kidney disease morbidity, the exact estimates of PIM appear to be a transitory “moving target” depending on where individual patients are on the integrated renal disease management pathway (CKD, Pre-dialysis, Dialytic, and post Kidney transplant) (MacRae et al., 2021). Since polypharmacy has evolved as the next “pit-stop” on the relentless therapeutic journey to downstream consequences such as ADRs and bidirectional interactions, restricting its definitional threshold to only disease-specific medications will assist in identifying patient populations who are more likely to benefit from its intervention strategies. Additionally, such change is likely to clarify the exact link between PIMs and polypharmacy.

## 2 Discussion and future perspectives

The apparent lack of determinative value in our current definition of polypharmacy in patients with CKD is made glaringly obvious by Wang et al. (2020)’s report on the proportion of patients with this therapeutic morbidity in their study cohort. Out of a total study population of 186 hospitalized patients, 98.4% of them satisfied the criteria for the definition of polypharmacy (≥5 medications). The additional difficulty of this definitional threshold is made more apparent by the fact that both the lower and upper limits of the Interquartile range of the average number of medications per patient in this study were 10–15 (clearly numerically higher than the definitional threshold of ≥5) (Wang et al., 2020). This meant that a definitional threshold of ≥5 medications cannot objectively identify the CKD patient population that could best benefit from polypharmacy-targeted interventions. The drug census of a typical Patient with CKD often includes a combination of drugs used in the management of the primary disorder; drugs for guideline-directed management of CKD itself; drugs for the management of the patient’s other morbidities. It is therefore obvious that for most patients the sum total of these “necessary” medications will be beyond currently suggested definitional thresholds (≥5 medications). The proportion of patients with PIM amongst the T2DM CKD cohort in Wang et al.’s study approximates estimates already reported. Unlike other organ systems, the kidneys and liver are particularly vulnerable to the effects of both polypharmacy and PIM due primarily to their role in drug metabolism.

## 3 Outstanding challenges of PIM and polypharmacy in CKD patients

### 3.1 Complex drug regimens

This represents the “inevitable fraction” of medication challenges CKD patients often have to endure. Complex medication regimens invariably include primary medications for managing CKD and its associated morbidities, as well as drugs used for the treatment of the primary kidney disease disorder. The consequence of this remains the resulting challenges in identifying and managing drug-drug, drug-food interactions, and downstream adverse effects. Although it must be stated that complex medication regimens in themselves may not be deleterious in the long term, rather what future studies should focus on are the morbidity and mortality-defining components of these regimens which may potentially be amenable to intervention.

### 3.2 Limited data on medication safety and efficacy

Currently, reliable data on the safety and efficacy of medications in CKD patients is limited, more so in patient cohorts with advanced CKD or those on dialysis. This stems often from tight and sometimes “discriminatory” exclusion criteria mix of randomized controlled trials (RCT) resulting in the exclusion of these patients from these studies. As previous studies have shown, indications of drugs adjudicated by these RCTs often get expanded *post hoc* to include patients with CKD and other morbidities. This then inevitably drives some of the unexpected adverse events resulting from exposure to these agents during phase IV monitoring. Because exposure to these drugs was not monitored under the usually intensive regimen of clinical trials, the signal for potential harms may continue unrecognized for a long time with the potential risks of therapeutic morbidities and preventable mortality. Until there is enduring regulatory intervention regarding the rising prevalence of posthoc expansion of drug indications in CKD patients, there is a need for more robust monitoring and strengthening pharmacovigilance practices amongst Renal and General physicians superintending over the prescribing traffic of these patients. This is vital in the early identification, assessment, and management of these events before irreparable harm ensues (Crescioli et al., 2021).

### 3.3 Frailty and other patient factors

A combination of patient-specific factors very often acting wholly significant impact on ability to both adhere to and deal with consequences of rising medication counts in patients with CKD. These often take form of sub-optimal medication adherence, worsening cognitive impairment. The latter is both a factor of declining kidney function itself as well as incident on it due to overall frailty. These factors generally makes it challenging to manage medications in CKD patients.

## 4 Conclusion

Potentially inappropriate medications maintain a bidirectional relationship with polypharmacy, and both share similar unwanted downstream consequences. Restricting the definitional threshold of polypharmacy in chronic kidney disease patients regardless of underlying primary etiology to only Kidney specific medications is key to reliably identifying patient cohorts for directed interventions. To compliment this, and additionally optimize the management of PIMs and polypharmacy in CKD patients, utility of established structural therapeutic frameworks such as pharmacotherapy optimization and or medication therapy management (MTM) may also prove invaluable long term.
